# The multiple roles of C-type lectin receptors in cancer

**DOI:** 10.3389/fonc.2023.1301473

**Published:** 2023-11-28

**Authors:** Qiao Li

**Affiliations:** Tongji Hospital, Tongji Medical College, Huazhong University of Sciences and Technology, Wuhan, Hubei, China

**Keywords:** C type lectin, cancer cell glycolation, immune response, bacterial infection, tumor microenvironment

## Abstract

C-type lectin receptors are a family of immune response receptors that can bind with a broad repertoire of ligands. It can function as innative immune receptors to surveillance bacteria, fungi, and virus invasions. The expressions of C-type lectin receptors (CLRs) are found in different types of tumors. But the role of C-type lectin receptors in cancer is not fully elucidated. And the underlying mechanisms of CLRs in carcinogenesis and tumor development remained unknown. It is known that CLRs bind to the glycosylated antigen on the cancer cells, regulating cancer cell invasion, migration, and metastasis. Meanwhile, the recognition of tumor glycans by antigen-presenting cells can stimulate antitumor immune response and induce immune tolerance. Also, some types of CLRs can be used as diagnostic markers for tumor cells, suggesting that C-type lectin can function as a new tumor therapeutic target and potential biomarkers. Given the therapeutic potential of CLRs in tumor treatments and the emerging roles of CLR in the tumor hallmarks, the multiple roles of CLRs in cancer were summarized in this review.

## 1 Introduction

C-type lectin receptors (CLRs) are a group of innate immune receptors expressed on antigen-presenting cells (APCs), including dendritic cells (DCs), Langerhans cells (LCs) and macrophages (1). CLRs mediated multiple functions of APCs including antigen presentation (1), T-cell priming against tumor or pathogen antigens (2). The role of C-type lectin (CLEC) in recognizing pathogens has been long recognized (3–9). However, the role of CLECs in cancer has not yet been fully elucidated. As major immune players, CLRs are involved in multiple tumor immune responses. They recognize glycosylated tumor–associated antigens, priming DC maturation and activation and inducing an active T-cell response (10). Tumor cells can also target CLECs to evade immune surveillance (10). Targeting antitumor vaccine to CLRs expressed on APCs has emerging as a potential strategy of vaccine development (2). In the present review, we introduced the family of CLRs and elucidated the multiple roles of CLEC in tumor biology.

## 2 Composition of CLEC family

Conserved pathogen-associated molecular pattern molecules can be recognized by host pattern recognition receptors (PRRs), the most well-known being Toll-like receptors (TLRs) and C type lectin receptors (CLRs) (11). CLRs are among the important PRRs associated with native immunity (3). There are two carbohydrate recognition domains (CRD) in the C-type lectin receptors, one can bind with mannose and N-acetylglucosamine (GlcNAc), the other one recognizes N-acetylgalactosamine (GalNAc) (12). Glu-Pro-Asn (EPN) tripeptide motifs containing CLRs bind with GlcNAc ligand and mannose, e.g., DC-SIGN. Glu-Pro-Asp (QPD) containing CLRs bind with GalNAc and galactose. Binding of the C type lectin receptors with ligands can activate the tyrosine-based activating motif (ITAM) signaling, recruit the tyrosine kinase and lead to the activation of downstream NF-kB activation, and active immune response (13). On the other hand, activation of immunoreceptor tyrosine-based inhibitory motif (ITIM)-containing CLRs, can recruit tyrosine phosphatases Src-homology-2-domain-containing protein tyrosine phosphatase 1 (SHP-1) or SHP-2, and negatively regulate immune response (13). CLR-mediated downstream signal transduction can be mediated by ITAM-containing adaptor proteins e.g., Fc receptor g chain (FcRg).

**Figure 1:**
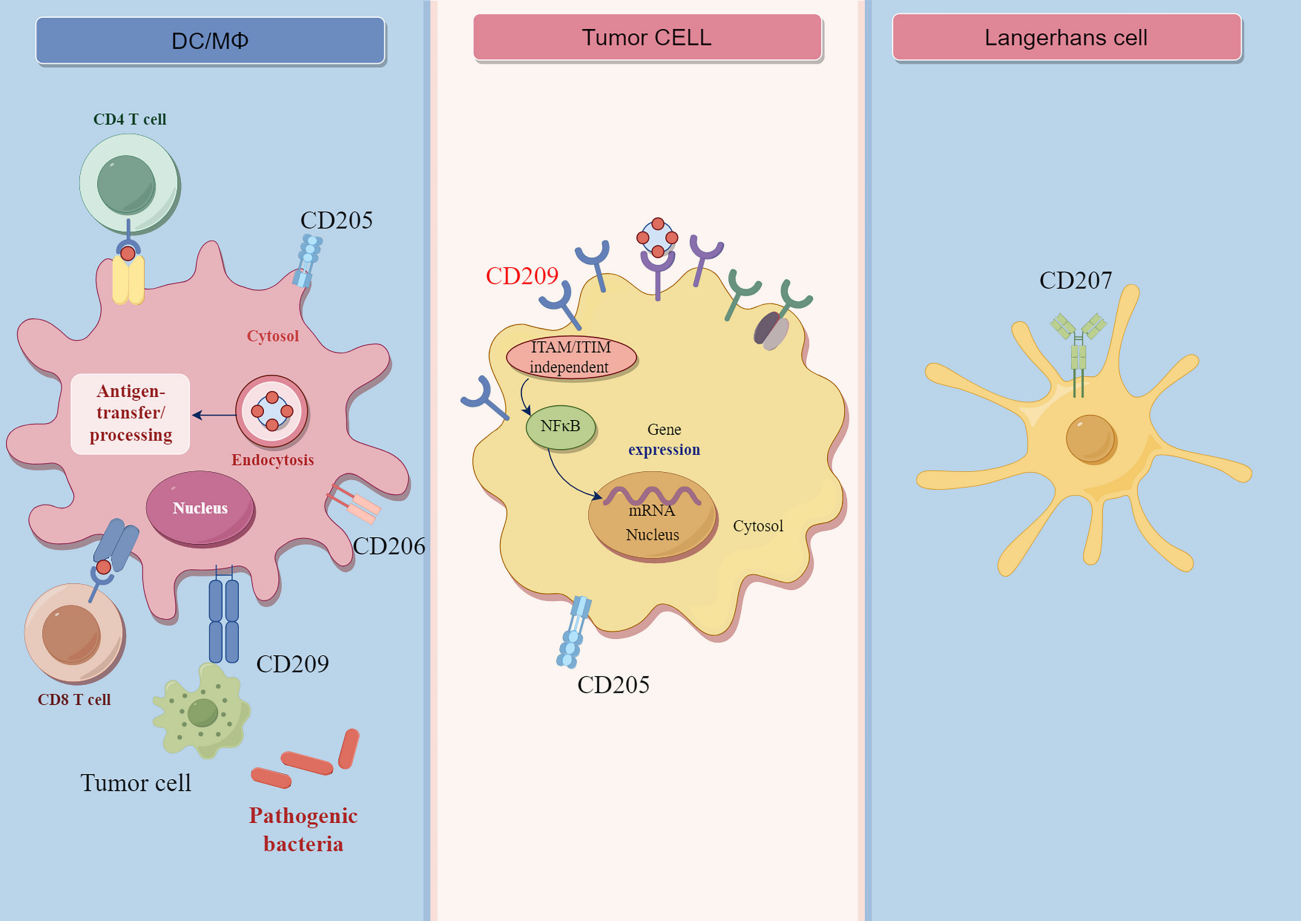
The structure and functions of CLRs. CD209 is expressed on macrophages and dendritic cells, and it is ITAM/ITIM independent receptor. The CD209 receptor can bind with carbohydrate ligands of pathogens or tumor cell antigen. The binding of CD209 receptor on tumor cells can lead to the activation of downstream NF-γB signaling. CD207 is mainly expressed by Langerhans cell, CD205 is expressed on macrophages and dendritic cells, myoepithelial cells, and cancer cells. CD206 is mainly expressed on macrophages and dentritic cells.

CLEC family members, such as dendritic cell-specific ICAM-grabbing non-integrin (DC-SIGN), CD206, and langerin (CD207), are highly expressed by DCs and phagocytes (14). Most CLRs expressed as membrane proteins are present on APCs (15). Hence, we discussed the expression of CLECs on APCs and tumor cells (Figure 1).

**Figure 2:**
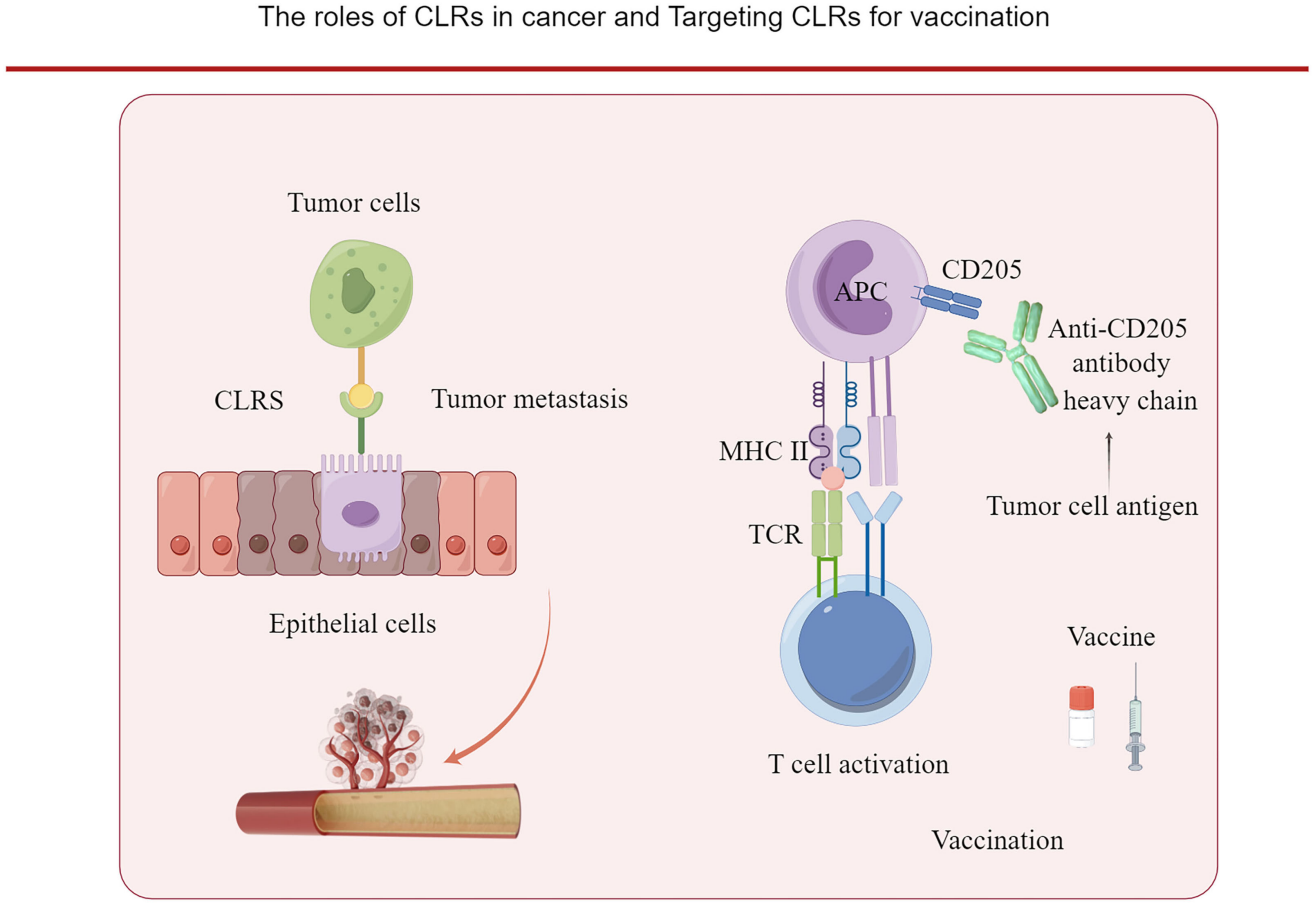
The roles of CLRs in cancer. The binding of CLRs with the carbohydrate ligands of cancer cells can promote the dissemination of the cancer cells. And CLRs can be targeted by vaccine for priming the T cells. Tumor antigen is cloned to the heavy chain of anti- CD205 antibody and targeted to dendric cells which express CD205, and initiate an active T cell response.

### 2.1 CD205

CD205 is a 205-kDa type I cell-surface protein that belongs to the CLEC family (16). CD205 is expressed on DCs and alveolar macrophages (17). CD205 is also expressed on tumor cells (16, 18).

### 2.2 CD206

Macrophage mannose receptor (MR, CD206) is a carbohydrate receptor belonging to type I CLECs (19–21). The MR binds to glycoconjugates terminated in mannose, fucose, or GlcNAc in a calcium-dependent manner (22, 23). It is mainly expressed in liver and spleen endothelial cells, in macrophages, and to a lesser extent, in DCs (24). The MR-binding receptor requires a partner to trigger phagocytosis in specialized cells such as macrophages (25). MR is also responsible for the recognition and phagocytosis of pathogens and allergens, promotion of Th2 immune responses, and antigen presentation (25).

### 2.3 CD207

CD207 is specifically expressed by Langerhans cells in the epidermis (26). Langerhans cells do not express CD209 (27). Although both CD207 and CD209 bind to HIV, they exhibit contrasting functions (26). Compared with CD209, CD207 is a barrier to HIV dissemination (28). Instead of promoting the dissemination of HIV, CD207 prevents HIV-1 transmission (28, 29). CD207 is also expressed on tumor tissues (30). One third of the primary breast tumors are positive for CD207, which is a marker of immature DCs or Langerin cells (31).

### 2.4 CD209

The DC-specific intercellular adhesion molecule-3–grabbing nonintegrin (DC-SIGN, CD209) is a type II integral membrane protein expressed on DCs and some tissue macrophages (32). DC-SIGN is mainly expressed on immature monocyte-derived DCs, and the maturation of the DCs decreases the expression of DC-SIGN (10)}. As an ITAM or ITIM independent receptor, activation of DC-SIGN leads to the activation of serine and threonine kinase Raf-1 and acetylation of the NF-κB subunit p65 (33). CD209/DC-SIGN is also a cell adhesion molecule expressed on APCs (32). DC-SIGN is expressed on the mucosal surfaces of fibrous connective tissue (34). It binds to human immunodeficiency virus (HIV) (14, 35, 36) and multiple pathogens (4, 5, 7, 37).

## 3 The roles of CLEC in cancer

### 3.1 CD205

Besides APCs, the thymic cortical epithelial cells express CD205, especially in thymic epithelial neoplasms, which can be used as a diagnostic marker (18). CD205 is also expressed in non-small cell carcinomas of the lung, squamous cell carcinoma of the head and neck, and squamous cell carcinoma of the esophagus (18). The expression of CD205 was detected in ovarian cancer and modulate metastasis (38). But the functional role of CD205 expression in some tumor types, including squamous cell carcinoma of the head and neck and non-small cell lung carcinoma, need further investigation (18). The CD205^+^ polymorphonuclear myeloid-derived suppressor cells (MDSCs) can promote tumor suppression (30).

### 3.2 CD206

CD206 is also upregulated in acute myeloid leukemia (39) and in the alveolar lavage fluid of patients with small cell lung cancer (40). It is detected in hepatocellular carcinoma (41), and its expression correlates with lower overall survival and disease-free survival (41).

### 3.3 DC-SIGN(CD209)

Human DC-SIGN can be expressed on not only APCs but also epithelial cells (42). Human cancer cells can express C-type lectin *in situ* (34, 41, 42). The expressions of DC-SIGN are reported on multiple tumor cells, including colon cancer (43, 44), gastric cancer cells (45), regulating tumor cells proliferation, migration, and metastasis (45, 46). Binding of DC-SIGN with colorectal cancer cell glycosylated antigen promote the secretions of IL-6 and IL-10, and induce an immune tolerogenic microenvironment (47). Many cancer cells, including colon and Lewis lung cancer cells, can express human DC-SIGN (46–48). It is expressed at a high level in metastatic colorectal cancer cell lines (46). The Binding of DC-SIGN on DC with tumor-specific glycosylation can suppress DC functions and felicitate immunosurveillance of the tumor cells (47, 49, 50). Fan et al. reported that CLEC promoted glioblastoma formation by regulating Phosphoinositide 3-kinase (PI3K)/V-akt murine thymoma viral oncogene homolog (AKT) signaling (51). CLEC was expressed on the colorectal mucosal surfaces (42). Jiang et al. reported the expression of DC-SIGN and DC-SIGNR in immunohistochemical assays of cancer tissues but only a weak expression in normal tissues (44). In contrast, the serum levels of DC-SIGN were higher than those in healthy controls (44). High numbers of DC-SIGN^+^ dendritic cells were also found in the lesions of cutaneous T-cell lymphoma (52).

The single-nucleotide polymorphisms in the DC-SIGN gene-encoding region were associated with the susceptibility of multiple cancers, for example, nasopharyngeal carcinoma (53) and colorectal cancer (54). Lu et al. found that single nucleotide polymorphisms (SNPs) in three C-type lectin genes, CD209, MBL2 and REG4, correlates with colorectal cancer (CRC) risk (54). It indicated DC-SIGN can function as biomarkers for the early diagnosis of cancer and predict the clinical outcome of malignant disease (50).

## 4 Roles of CLEC in cancer

CLR acts as an antigen-presenting receptor during antigen capture and presentation (55). DC-SIGN can recognize the foreign glycans on the parasite and bacteria in a Ca^2+^-dependent manner (9, 14, 35, 36). Except for the antigen presentation function of CLEC, CLRs can also recognize and bind with the glycosylated proteins in a Ca^2+^-dependent manner (56). Multiple types of tumor antigens can be recognized by CLR (57). Lewis antigen, N-acetylgalactosamine, and glycans, components of tumor cells, can bind to CLRs as ligands (10).

### 4.1 Glycosylation of CLRs in cancer cells

Glycosylation is one of the markers of cancer cells (58, 59). The glycosylation of the cancer cells is associated with the acquisition of other hallmarks of the cancer cells, including evading immune surveillance, invasion, metastasis, and so forth (60) Glycosylation refers to the linkage of saccharides to saccharides, proteins, or lipids (14). The change of glycosylation state of cancer cells is be attributed to the aberrant expression of glycosyltransferases, the localization of glycosyltransferases, the conformation of the peptide backbone (14). Glycosylation can affect the function of E−cadherin, a glycoprotein modulating epithelial cell–cell adhesion (14). Glycans can have a profound effect on the metabolism shift of cancer cells (14). High DC-SIGN and L-SIGN in B-cell ALL correlated with poor prognosis of the disease (10). In Non-Small Cell Lung Cancer (NSCLC), higher CD209^+^ M2 macrophages i is correlated with metastasis (2).There is also a positive correlation between the progression of colorectal cancer clinical stage and remote metastasis and beta-galactoside-specific lectin galectin-3 expression (15, 16).”

The glycosylation of tumor antigen during malignant transformation can promote the binding of the carbohydrate structures of tumor cells with C-type lectins on dendritic cells (47). The glycosylation of carcinoembryonic antigen (CEA) and CEA-related cell adhesion molecule 1 (CEACAM1) on cancer cells are two important examples (47). CEA is widely expressed in gastrointestinal cancers, including colorectal cancer (47). The CEA protein undergoes aberrant glycosylation during cancer progression, for example, in colon carcinoma (61–63). Following glycosylation, the CEA is recognized by DC-SIGN, but the nonglycosylated CEA is not recognized (64). Lea/Leb glycans are expressed at a high level on colon cancer epithelial cells, but not on normal colon epithelial cells (47). The expression of Lewis blood group family of antigens during malignant transformation increases (65). Lewis X and *de novo* Lewis Y on tumor-specific CEA in intestinal epithelial cells (IECs) increase during the carcinogenesis (43). Lewis antigens can bind with DC-SIGN and induce the secretion of inflammatory cytokine secretions (e.g., IL-6 and IL-10) which can promote the establish of a tolerogenic microenvironment for colorectal cancer (49).

Malignant transformation increases the glycosylation of the cancer cell (66). CEACAM1 is highly expressed in ovarian cancer (67). DC-SIGN can bind with the high-mannose oligosaccharides in Follicular lymphoma (68). It can mediate the binding of DCs and colorectal cancer cells *in situ* (47). MUC1 is a highly glycosylated tumor antigen that binds to CLEC. It is expressed in breast cancer and undergoes glycosylation during malignant transformation (69, 70).

### 4.2 CLRs mediates escape of immunosurveillance

DCs and macrophages are two important components in the induction of antitumor immune responses (49). Van Kooyk reported that DC-SIGN on immature DCs instead of mature DCs could recognize the glycosylated CEA on colorectal cancer cells (49). This interaction is mediated by the binding of CEA-carrying Lewis^X/Y^ on colorectal cancer cells with DC-SIGN on DCs (49). This interaction does not exist between DC-SIGN and CEA with low levels of Lewis antigen on the normal colon cells (49). This might contribute to the escape of immunosurveillance by colon cancer cells (49).

### 4.3 CLRs promote metastasis of cancer cells

The breast cancer cells express clusterin, which undergoes aberrant fucosylation and interacts with DC-SIGN (71). The glycan in the tumor cells can also bind to CLEC, resulting in metastasis (43). The colon cancer cells bind with DC-SIGNR on liver sinusoidal endothelial cells and promote the migration of colon cancer cells to liver (43) (Figure 2). The expression of metallothioneins and Matrix metallopeptidase 9 (MMP9) which can degrade extracellular matrix in colon cancer cells are regulated by DC-SIGNR (43). In follicular lymphoma, the expression level of glycosyltransferases, which promote the synthesis of both N- and O-linked oligosaccharides, changed, leading to the aberrant glycosylation of the tumor cells, and benefiting the tumor cell migration and metastasis (72). And the expression of DC-SIGN on the lymphatic endothelial cells can potentially promote the metastasis of follicular lymphoma (73, 68). L-SIGN expressed by lymphatic endothelial cells can bind with high-mannose glycans on malignant follicular lymphoma B cells, and promote the dissemination of follicular lymphoma (73).

DC-SIGN can promote the metastasis of colorectal cancer through the PI3K/Akt/β-catenin signaling pathway and further upregulation of the transcriptions of MMP-9 and VEGF (46). CRC metastases are facilitated by DC-SIGN *in vitro* and *in vivo*. Tyrosine-dependent signaling is activated by DC-SIGN, and activate PI3K/Akt/β-catenin signaling which is tumor promotive (46). Platelet-activating C-type lectin-like receptor-2 (CLEC-2) can promote the metastasis of hematogenous tumor and facilitate tumor progression (74). Hematogenous metastasis is enhanced by tumor cell-induced platelet aggregation which is mediated through CLEC-2–podoplanin interaction.

### 4.4 CLRs mediate the edit of tumor microenvironment

Tumor cells can polarize the macrophages to a phenotype that facilitates metastasis (48). The M1 and M2 macrophages are acquired by macrophages polarized by interleukin (IL)-10 and IL-4/IL-13, respectively (75). DC-SIGN is found to be expressed on tumor immunosuppressive M2 macrophages (75), and the expression of DC-SIGN can be induced by IL-4 and macrophage colony stimulating factor (M-CSF), indicating that DC-SIGN is a marker of M2 macrophages (75). Therefore, it indicated that DC-SIGN contributes to an immunosuppressive microenvironment (76). For example, Lewis lung cancer cells can secrete IL-4 to polarize the macrophages to M2 phenotypes which express DC-SIGN and facilitate immune evasion (48).

Tumor-associated macrophages (TAMs) occupy 5%–40% of the tumor tissues (77), and their abundance correlates with poor prognosis (78). CD206, another member of CLRs, can modulate the tumor environment (79). It is not expressed in classical M1 macrophages and only in M2 macrophages, which secrete cytokines interleukin (IL)-4, IL-13, and IL-10 (80). Enninga et al. found that carbohydrate-binding protein galectin-9 bound to CD206 on the macrophages and induced tumor formation (79). Haque et al. found that CD206^+^ tumor–associated macrophages are present in oral squamous cell carcinoma (OSCC) (81). The coculture of OSCC cells with CD206^+^ cells promote their proliferation and invasion, this is due to the epidermal growth factor (EGF) produced by CD206^+^ TAMs (81).

The skin lesions of cutaneous T-cell lymphoma also express CLRs, including CD206, CD207, and CD209 (52). The expression of CD209 is correlated with poor prognosis in acute lymphoblastic leukemia (82). DC-SIGN expressing TAMs is associated with an immunosuppressive tumor environment (83). The inhibition of DC-SIGN-expressing TAMs using a neutralizing antibody can reactivate the antitumor immunity and improve the immunotherapy against bladder cancer (83).

### 4.5 Role of CLEC-mediated infection in cancer

The relationship between chronic infection and tumor development has been recognized, e.g., in colorectal cancer (CRC) (13), *Helicobacter pylori* infection in developing gastric cancer (84). The role of microbiota in the development of CRC is under extensive investigations (13). The interaction between microbiota and carcinogenesis is characterized in breast cancer (85), CRC (86), gastric (87), lung cancer (88), bladder cancer (89) and multiple tumor types (90). C type lectin can play a role in mediating the bacteria infection and tumor development (13).

The three major functions of DC-SIGN include T-cell priming, regulation of DC migration, and antigen presentation (34). The CLEC also acts as a pathogen recognition receptor (34). CLRs are important receptors on pathogens that mediate the interaction between the host and pathogens (34). It is widely accepted that CLEC mediates the binding of pathogens to epithelial cells and is involved in complement-mediated opsonophagocytosis (91). It was also demonstrated that DC-SIGN interacted with bacterial pathogens such as *Mycobacterium tuberculosis* (92) and *Helicobacter pylori* (93) and other bacteria (91).

Emerging evidence suggests that, besides the canonical function of CLEC in bacterial adherence, macrophages expressing the CD209 receptor participates in bacterial persistent infection (94). Macrophages and dendritic cells can function as a shelter of persistent bacterial infection (95). Inflammation caused by the persistent infection of bacteria can favor the carcinogenesis of cancer (96). Release of cytokines and chemokines, remodeling an immune suppressive microenvironment, damage to the DNA function to promote the carcinogenesis and facilitate tumor development (97).

## 5 CLEC as potential therapeutic targets and future perspective

As a pathogen recognizing receptor of various pathogens, CLRs can recognize HIV (14), *Mycobacterium* species, *Helicobacter pylori* via mannose or fucose moieties (93). Mucin 1 (MUC1), as a transmembrane mucin glycoprotein on the epithelial cells, can bind with multiple bacteria, e.g., *H. pylori*, *Pseudomonas aeruginosa*, *Salmonella typhi* and *Escherichia coli* (98). The expression of MUC1 on macrophage can be upregulated by *Pseudomonas aeruginosa* and function in host defense against bacterial infection (98). Binding of MUC1 can lead to the activation of NF-κB signaling and the nuclear translocation of MUC1–p65 complex which can upregulate EMT master modulator Zinc finger E-box binding homeobox 1 (ZEB1) (98).

C type lectins have been implicated in the intestinal microbiota-inflammation-cancer axis (13). NF-κB is essential downstream signal of CLRs, and connect inflammation and carcinogenesis (13). Microbiota is emerging as a critical regulator in tumor development. Microbiota dysbiosis take part in the carcinogenesis of colon cancer by inducing hypermethylated genes to cause epigenetic regulation (13). The metabolites of microbiota can cause intestinal inflammation (13).

Given the role of persistent bacterial infection in tumor development, C type lectin mediated bacterial infection is potentially involved in tumorigenesis. Further investigations are warranted to elucidate the potential roles of CLRs in carcinogenesis and their roles as therapeutic targets of tumors. Small-molecule inhibitors that inhibit CLEC–ligand interaction can overcome pathogen infections, such as HIV, mediated by CLEC (99). Synthetic glycodendrimers can block HIV transmission via competitive inhibition through DC-SIGN on DCs (100). Lewis X component from human milk can inhibit HIV-1 transfer to CD4+ T lymphocytes by binding with DC-SIGN (101). Especially, the carbohydrate structures, such as Lewis antigen, ligands of CLRs are shared by pathogens and tumors. It indicated blocking the CLRs on tumor cells might inhibit the implications of CLRs in tumor biology.

On the other hands, targeting antigens to CLRs can stimulate immune response to tumor cells (10). Scodeller et al. has investigated tumor therapies using the CD206-binding peptide target tumor cells (102). Lepland et al. found the CD206-binding mUNO peptide coupled with molecular and nanoscale cargoes can interact with mouse CD206 (103), and can target M2 TAMs in breast cancer (103). These inspiring finding encourage the investigation of the therapeutic potential of CLEC as inhibitor targets.

The increase of Lewis X and Lewis Y on CEA enhance the interaction between DC and intestinal epithelial cells, and mediate tumor cells to escape immunosurveillance (49). The interaction between aberrantly glycosylated CEA and CEACAM1 suppress the function and differentiation of monocyte-derived dendritic cells by secreting immunosuppressive cytokines IL-6 and IL-10 (57). It indicated the molecular basis of inhibiting the binding of C type lectin with tumor glycosylated antigen in antitumor therapeutic strategies.

Also, antitumor vaccine developed by conjugating human cancer antigen with anti-CD205 antibody opened a potential field of tumor vaccine investigation (104, 105). CD205 targeting vaccine has been initiated by Ralph M. Steinman (104). Human cancer antigen mesothelin were conjugated with antibody targeting mouse DEC-205 receptor (104). Stronger CD4+ T-cell responses and humoral immune responses were induced (104). Monoclonal anti-C type lectin receptor antibodies were engineered to express as vaccine proteins (104). It improved the improving the delivery of human cancer antigen to dendritic cells (104). Naïve CD4+ CD25-Foxp3- T cells were converted into stable Foxp3+ Treg cells favoring by dendritic cells.

Except for the application development in the antitumor vaccine development, CLRs targeting vaccine is also explored in anti-HIV infection vaccine and pulmonary mucosal immune responses against pneumonic plague (106, 107).

In conclusion, a better understanding of the interaction between CLEC and tumor cells may contribute to the development of new antitumor strategies. C type lectins are emerging as a new target for treatment of cancer. Understanding the complex roles of C-type lectins in tumor will initiate new dimensions of anti-tumor strategies.

## Author contributions

QL: Writing – original draft, Writing – review & editing.
